# Assessment of Adaptation Status of Reintroduced *Equus Przewalskii* Based on Comparative Analysis of Fecal Bacteria with Those of Captive *E. Przewalskii*, Domestic Horse and Mongolian Wild Ass

**DOI:** 10.3390/ani12202874

**Published:** 2022-10-21

**Authors:** Dini Hu, Chen Wang, Make Ente, Ke Zhang, Dong Zhang, Xuefeng Li, Kai Li, Hongjun Chu

**Affiliations:** 1 School of Ecology and Nature Conservation, Beijing Forestry University, Qinghua East Road No.35, Beijing 100083, China; 2 Xinjiang Kalamaili Ungulate Nature Reserve Management Center, Changji 831100, China; 3 Xinjiang Research Centre for Breeding Przewalski’s Horse, Urumqi 831700, China; 4 Institute of Forest Ecology, Xinjiang Academy of Forestry, Urumqi 830063, China

**Keywords:** environmental adaptation, *Equus przewalskii*, intestinal microbiota, reintroduced animal, wild animal

## Abstract

**Simple Summary:**

Reintroduced Przewalski’s horse (RPH) has been released into the wild for 20 years, but their viability is still far away from that of sympatric wild animals. At present, RPHs cannot easily cope with climatic change, diseases and disturbance of other animals and need to be captured in the winters to be dewormed to increase their survival rate. Intestinal microbiota have been studied and are considered to be an important indicator to reflect the health status of the host. Thus, the differences in intestinal bacterial community composition and structure among RPHs, captive Przewalski’s horses, sympatric domestic horses and sympatric Mongolian wild asses were analyzed in this study, which can help to evaluate the adaptability of RPH and provide important reference information for further development of protection strategies.

**Abstract:**

Intestinal microbiota play an important role in the survival of the host. However, no study to date has elucidated the adjustment of intestinal microbiota of the host during rewilding. Thus, this study aims to describe the intestinal bacterial community of reintroduced Przewalski’s horse (RPH) after being released into their original habitat for approximately 20 years in comparison with that of captive Przewalski’s horse (CPH), sympatric domestic horse (DH) and Mongolian wild ass (MWA) by sequencing the 16S rRNA gene. The results showed that the prevalent bacterial communities were different among CPHs, RPHs, DHs and MWAs at the family level. NMDS and ANOSIM analysis showed that the pattern of bacterial community composition in captive equines was distinct from that in the wild groups. It is shown that some bacteria had significant differences among different taxa (*p* < 0.001), such as *Firmicutes*, *Bacteroidetes*, *Armatimonadetes*, *Clostrida*, *Bacteroidia*, *Clostridiales*, *Bacteroidales*, *Rikenellaceae* and *Bacteroidales*_UCG-001. These bacteria were associated with the transition from in captive to in the wild (CPH and RPH), which reflected the change of environmental conditions. Meanwhile, *Proteobacteria*, *Clostridia*, *Bacilli*, *Negativicutes*, *Gammaproteobacteria*, *Clostridiales*, *Bacillales*, *Selenomonadales*, *Pseudomonadales* and *Planococcaceae* were the changed groups among RPHs, MWAs and DHs, which are related to feeding habits and diseases. Our results clearly showed the differences between intestinal microbiota in reintroduced animals and wild animals and led us to understand the survival state of reintroduced animals in the wild.

## 1. Introduction

**Figure 1:**
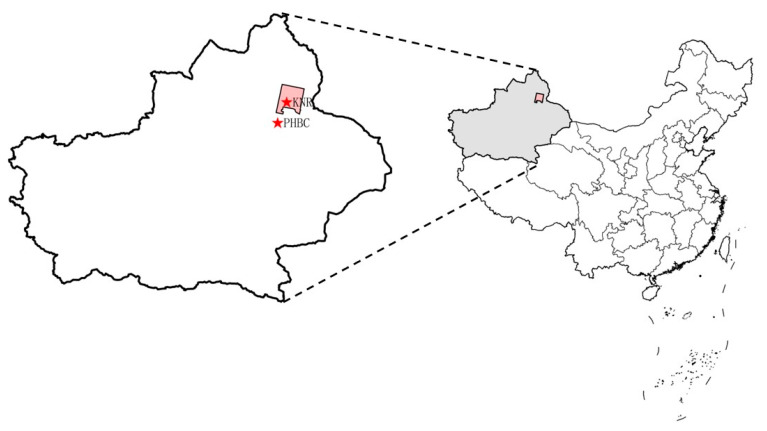
The location of KNR and PHBC. The figure was generated by Google Maps.

Being the last wild equid in the world, *Equus przewalskii* is classified as “EN” by the International Union for Conservation of Nature (IUCN) [1]. The natural population of *E. przewalskii* became extinct in the wild in the 1960s, and all of the extant captive population in the world are the offspring of 13 captured wild *E. przewalskii* that are bred in European and American zoos or ranches [2]. To save this endangered animal, the Foundation Reserves Przewalski Horse developed a population reintroduction program. Accordingly, horses from Europe and America were imported to the Przewalski Horse Breeding Center (PHBC) in Xinjiang, China, in 1985 [3] (Figure 1). In 2001, the enlarged species were released into the adjacent Kalamaili Nature Reserve (KNR) with an area of approximately 13,000 km^2^ [4] (Figure 1), which resulted in the formation of three equine species living in the same region: reintroduced Przewalski’s horse (RPH), Mongolian wild ass (MWA) and domestic horse (DH) [4]. By April 2021, the population of RPHs had increased to 267. The previous observations on the behavior of RPHs showed that there have been significant changes in the behavioral patterns of feeding, mating, water source utilization and stress response [5,6,7,8,9,10]. However, RPHs are still far away from wild animals in that they cannot easily cope with climate change, diseases and disturbance of other animals [11,12]. At present, RPHs have to be captured in winter to be dewormed of parasites (*Gasterophilus* sp.) to increase their survival rate. Having been released into the wild for 20 years, *E. przewalskii* has attracted intense attention to their living state and environmental adaptability [13,14].

With the development of molecular sequencing technology, intestinal microbiota have been studied and are considered to be one of the important indicators to reflect the health status of the host [15,16]. Large numbers of microbes live in the intestines of animals and form a complex micro-ecosystem [17,18]. The coexistence between the host and intestinal microbes can promote a healthy animal, even among reintroduced and relocated animals [19]. In contrast, a disorder or imbalance of intestinal microbes has adverse effects on the host [20,21]. A growing number of studies have shown that the intestinal microbial community directly participates in physiological processes such as metabolism, immunity, endocrinology and nerve activity [22,23,24,25,26]. In addition, a study has indicated that the reintroduction process can increase the intestinal bacterial diversity and significantly change the abundance of *Proteobacteria* [27]. This might be an effective strategy to monitor the adaptation of reintroduced animals to their new environments because DNA is directly extracted from feces and followed up with sequencing and microbial identification without the need for handling or capturing animals in the wild.

Reintroduced animals need to adapt to the environment after being released from captivity into the wilderness, due to the changes of environmental conditions. The biggest difficulty for RPHs in the wild is that they need to compete with other wild animals for optimal food resources. Thus, the most important factor is feeding habits. The objective of this study was to evaluate the adaptability of *E. przewalskii* and provide reference information for further development of protection strategies, because the effect of feeding habit transitions on intestinal microbiota, when RPHs are released to the wild, has not been studied yet. Therefore, we decided to describe the intestinal bacteria of *E. przewalskii* in two different states (in captive and in the wild) and three sympatric equine species living in KNR (RPHs, MWAs and DHs), aiming to discover the characteristics of intestinal microbiota of RPHs and their differences between those of captive Przewalski’s horses (CPHs) and native wild animals (DHs and MWAs).

## 2. Materials and Methods

This study was carried out in accordance with Chinese laws, regulations of the Beijing Forestry University and guidelines for animal research [28]. The experimental protocol was reviewed and approved by the Institution of Animal Care and the Ethics Committee of Beijing Forestry University. The management authorities of the Kalamaili Nature Reserve and Przewalski Horse Breeding Center approved the collection of Przewalski’s horse fecal samples. 

### 2.1. Study Area

The KNR and PHBC were selected as the sampling sites (Figure 1). The KNR (44°36′~46°00′ N, 88°30′~90°03′ E), a desert steppe, is located in the Junggar Basin of Mongolia and Xinjiang, China, with an altitude of 600–1470 m. The average annual temperature is 2.4 °C, the annual precipitation is 160 mm, and the annual evaporation is 2000 mm [12,29]. The vegetation in the reserve is sparse and mainly consists of desert plants, such as small xerophytic trees, shrubs and drought-tolerant herbs, and the plant coverage is generally 20–30% [30]. The dominant species are *Stipa capillata*, *Ceratoides latens*, *Ceratoides arenarius*, *Anabasis brevifolia* and *Artemisia* spp. [31], which are the food sources of RPHs, DHs and MWAs. In addition, some animals live in the KNR, including nearly 50 wild animals of the EV class, such as RPHs and MWAs, as well as a small herd of DHs [4,32]. PHBC (45°49′~46°04′ N, 89°14′~89°36′ E) is located on the plains of northern Mount Bogda, the main peak of Tian Shan. The altitude is 570–585 m, and the total area is approximately 6,000,000 km^2^. CPHs are kept at PHBC, and alfalfa, corn flour and carrots and water are provided there [33,34].

### 2.2. Sample Collection

In June 2018, 10 adult RPHs (5 female, 5 male), 10 MWAs (5 female, 5 male) and 10 DHs (5 female, 5 male) were selected from the wild in KNR and kept individually in temporary enclosures, where the fresh fecal samples were collected. At the same time, 10 fecal samples of adult CPHs (5 female, 5 male) were collected from PHBC. These samples were used for intestinal bacteria analysis. The sex information of animals can be found in Table 1. The RPHs received the ivermectin treatment in winter; the half-life of ivermectin is about 3 days [35]. The animals did not receive the additional treatment of antibiotics or anthelmintics during the study. Fresh fecal samples were immediately collected in sterile centrifuge tubes after defecation. Then, the samples were sealed, labeled and put in the mobile refrigerator immediately after collection, until transported to the laboratory. The fecal samples were stored at −80 °C prior to DNA extraction.

### 2.3. DNA Extraction and 16S rRNA Sequencing

A Qiagen fast stool mini kit (QIAGEN Sciences, Dusseldorf, Germany) was used to extract the total bacterial DNA following the manufacturer’s protocol. The integrity of the nucleic acids was evaluated by electrophoresis on a 1.0% agarose gel containing ethidium bromide. A Qubit dsDNA HS Assay Kit (Life Technologies, Carlsbad, CA, United States) was used to determine the concentrations of extracted DNA. The extracted total DNA was stored at −80 °C.

The bacterial 16S rRNA gene fragments (V3-V4 region) were amplified using the universal bacterial primers 338F (5’-ACTCCTACGGGAGGCAGCA-3’) and 806R (5’-GGACTACHVGGGTWTCTAAT-3’) [36]. The PCR condition was set as follows: initial denaturation at 95 °C for 5 min, followed by 25 cycles of 95 °C for 30 s, annealing at 50 °C for 30 s, 72 °C for 40 s and 72 °C for 7 min. The PCRs were performed in triplicate, and the PCR products were mixed with the same volume of 2 × loading buffer and subjected to 1.8% agarose gel electrophoresis for detection. All of the PCR products with a bright main band of approximately 469 bp were chosen and mixed at equal ratios. Subsequently, the GeneJET Gel Extraction Kit (Thermo Fisher Scientific, Waltham, MA, United States) was used to purify the mixture of the PCR products. The Agilent 2100 Bioanalyzer (Agilent Technologies, Palo Alto, CA, United States) and Qubit 2.0 Fluorometer (Thermo Fisher, Waltham, MA, USA) were applied to validate and quantify the sequencing libraries, respectively. Finally, the Illumina HiSeq 2500 platform (Illumina, Inc., San Diego, CA, United States) by Biomarker Bioinformatics Technology, Co., Ltd. (Beijing, China) was used to conduct the paired-end sequencing. 

### 2.4. Data Analysis

The procedures for bioinformatic analysis of high-throughput sequencing followed those in Hu et al. (2021) [14]. Raw fastq files were demultiplexed and quality-filtered using QIIME 1.9.1 (http://qiime.org/install/index.html (accessed on 16 October 2022)) with the following criteria: (i) The 300 bp reads were truncated at any site receiving an average quality score <20 over a 50 bp sliding window, and the truncated reads shorter than 50 bp were discarded. (ii) Exact barcode matching, 2 nucleotide mismatch in primer matching and reads containing ambiguous characters were removed. (iii) Only sequences that overlap longer than 10 bp were assembled based on their overlap sequence. Reads which could not be assembled were discarded. Sequences were grouped into operational taxonomic units (OTU) using the clustering program VSEARCH 2.22.1 (https://github.com/torognes/vsearch/releases (accessed on 16 October 2022)) against the SILVA 138 database preclustered at 97% sequence identity, and chimeric sequences were identified and removed using UCHIME 4.1 (http://www.drive5.com/uchime/uchime_download.html (accessed on 16 October 2022)). The Ribosomal Database Project (RDP) classifier (version 2.11, https://sourceforge.net/projects/rdp-classifier/ (accessed on 16 October 2022)) was used to assign taxonomic categories to all OTUs at a confidence threshold of 0.8 [37]. The raw data have been checked for normality based on random subsampling (rarefying) [38,39]. QIIME2 was applied to calculate the indexes of alpha diversity and beta diversity for the analysis of bacterial community complexity in the fecal samples (https://qiime2.org/ (accessed on 16 October 2022)) [40]. The alpha diversity of the Shannon and Simpson indexes was used to reflect community diversity. The alpha diversity of Chao and Sobs was used to analyze the community richness. The Student’s t-test was applied to determine significant differences in alpha diversity between 2 groups (RPH vs CPH, RPH vs MWA, RPH vs DH, respectively). Nonmetric multidimensional scaling (NMDS), based on the unweighted UniFrac similarities of OTUs’ composition, was applied to rank the bacterial communities, and the analysis of similarity (ANOSIM) was applied in our study to determine the differences among equine animals using R software 4.2.2 (https://www.r-project.org (accessed on 16 October 2022)) [41]. The Kruskal–Wallis H test and Wilcoxon rank-sum test in STAMP 2.1.3 (https://beikolab.cs.dal.ca/software/STAMP (accessed on 16 October 2022)) were used to observe the significant differences between samples, and the *p*-value was tested by Bonferroni correction with the threshold set as 0.05. 

## 3. Results and Discussions 

### 3.1. Sequence Statistics

A total of 3,199,617 reads were obtained from 40 fecal samples, and 2,745,670 clean tags were retained after quality control and chimera removal. An average of 68,642 clean tags could be obtained from each sample. Good’s coverage was used to test the integrity of the sequences, the value of which was over 99.67% for all samples in this study, indicating that most bacterial species present in the samples had been detected. A total of 3153 OTUs were allocated, which could be annotated to 21 phyla, 32 classes, 68 orders, 116 families and 270 genera. 

### 3.2. The Differences in the Microbiota Diversity among Different Groups

**Figure 2:**
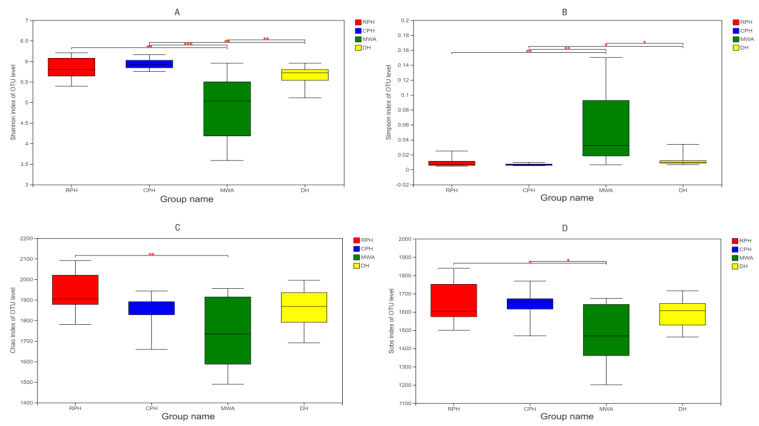
Box plot of alpha diversity indexes. (**A**) Shannon and (**B**) Simpson indexes reflect the diversity of OTU in samples. (**C**) Chao and (**D**) Sobs indexes reflect the OTU abundance in samples. The colors red, blue, green and yellow represent RPHs, CPHs, MWAs and DHs, respectively. *p < 0.05, **p < 0.01 and ***p < 0.001.

It was noted that the alpha diversity only showed significant difference between RPHs and MWAs (*p* < 0.05). The results indicated that captive Przewalski’s horses (CPHs) were more diverse than reintroduced Przewalski’s horses (RPHs) (Shannon: *p* = 0.311; Simpson: *p* = 0.210), and RPHs were more diverse than domestic horses (DHs) (Shannon: *p* = 0.128; Simpson: *p* = 0.359) and Mongolian wild asses (MWAs) (Shannon: *p* = 0.002; Simpson: *p* = 0.008) (Figure 2A,B). Furthermore, the results showed that RPHs have more richness than CPHs (Chao: *p* = 0.051; Sobs: *p* = 0.594), DHs (Chao: *p* = 0.108; Sobs: *p* = 0.173) and MWAs (Chao: *p* = 0.009; Sobs: *p* = 0.014) (Figure 2C,D). The results were consistent with previous studies showing that the difference in animal species is the main factor influencing the intestinal microbial alpha diversity and richness [42]. However, in contrast to a previous study, where the intestinal microbiota diversity in wild populations was usually more diverse and complex than in captivity [25], the captive status did not significantly affect the intestinal microbial alpha diversity in Przewalski’s horses [43,44].

**Figure 3:**
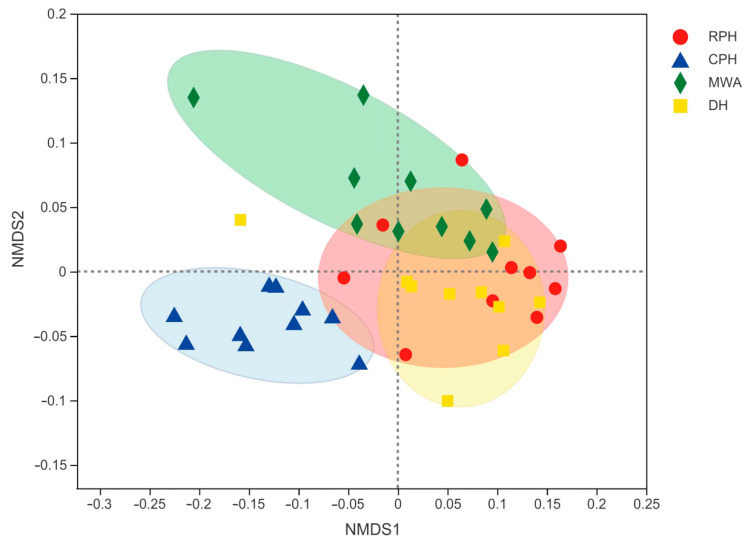
Nonmetric multidimensional scaling (NMDS) scatterplot represents the difference in intestinal microbial community composition among CPHs, RPHs, DHs and MWAs. The distance between points indicates the degree of difference based on the unweighted UniFrac. Data were from a study conducted in KNR and PHBC, China, during June 2018. CPHs were fed with alfalfa, corn flour and carrots. RPHs, DHs and MWAs freely picked *Stipa capillata*, *Ceratoides latens*, *Ceratoides arenarius*, *Anabasis brevifolia* and *Artemisia* spp. in the wild.

The NMDS analysis showed a similar composition within sympatric RPHs, DHs, and MWAs, but different from CPHs (Figure 3). The ANOSIM analysis revealed significant differences in bacterial communities between RPHs and CPHs (R = 0.7991, *p* = 0.001), but no significant difference between RPHs and MWAs (R = 0.1449, *p* = 0.043) and between RPHs and DHs (R = 0.0000, *p* = 0.413). In our study, CPHs were fed with high-nutrient alfalfa supplemented with a small amount of corn flour and carrots [45]. RPHs, DHs and MWAs freely picked and ate plants in KNR, including *Stipa capillata*, *Ceratoides latens*, *Ceratoides arenarius*, *Anabasis brevifolia* and *Artemisia* spp. [31]. Thus, the differences in bacterial community composition between wild and captive animals may result from the differences in diet. A similar result was also found by Liu et al. (2021) [46] that the bacterial beta diversity of the wild population was distinct from that of the captive population.

### 3.3. The Comparison of Microbial Community Composition and Structure

**Figure 4:**
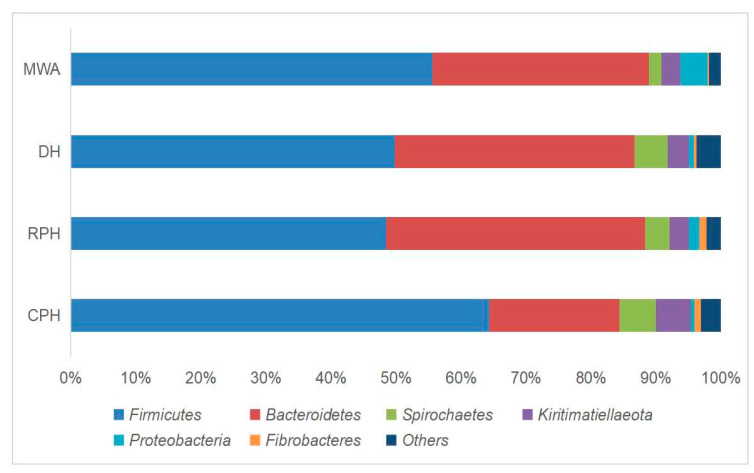
Top relative abundances of phyla, ranked by 1 and 0 orders of magnitude based on RPHs, with relative abundances of the corresponding phyla in CPHs, DHs and MWAs shown. Data were from a study conducted in KNR and PHBC, China, during June 2018.

**Figure 5:**
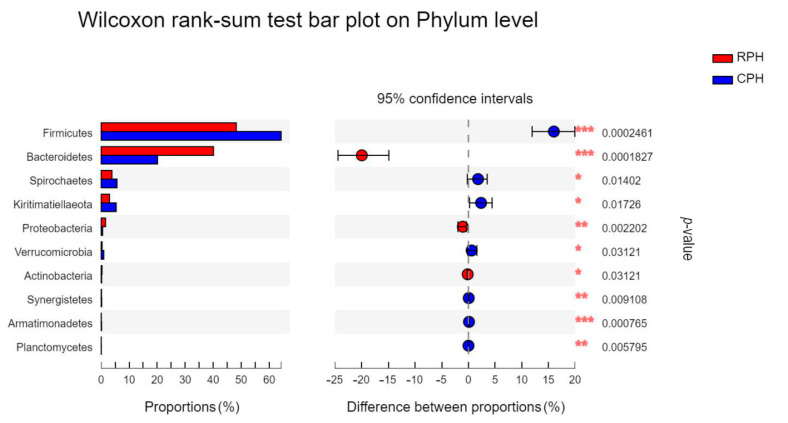
The bar plot shows the bacterial phylum with significant differences among the CPH and RPH. *p < 0.05, **p < 0.01 and ***p < 0.001.

At phylum level, *Firmicutes* (CPHs: 64.32%, RPHs: 48.45%, DHs: 49.81%, MWAs: 55.60%) and *Bacteroidetes* (CPHs: 20.02%, RPHs: 39.85%, DHs: 36.84%, MWAs: 33.33%) were the most prevalent phyla in all samples (Figure 4). The proportion of them was between 85% and 90% and was consistent with previous studies conducted on herbivores. These two phyla are the main group of microorganisms making up the intestinal microbiota of herbivores [47,48,49]. A study of *E. przewalskii* by Li et al. (2019) [42] also showed that *Firmicutes* is significantly higher in the captive group than in the wild group. As we know, *Bacteroidetes* are mainly responsible for metabolizing steroids, polysaccharides and bile acids, contributing to the absorption of polysaccharides and the synthesis of proteins in the host [50,51]. *Firmicutes* and *Bacteroidetes* have an excellent performance in the process of anaerobic digestion, and accordingly, the ratio of *Firmicutes* to *Bacteroidetes* (F/B ratio) is usually regarded as an indicator of this process [52]. Biddle et al. (2018) [53] found that the F/B ratio is positively related to obesity in horses. The results showed that CPHs had a higher ratio of *Firmicutes* to *Bacteroidetes* than RPHs, DHs and MWAs (Supplementary Table S1). As mentioned before, CPHs were fed by alfalfa and RPHs, DHs and MWAs freely picked and ate plants in the wild. In addition, the Wilcoxon rank-sum test showed that there is a significant difference in *Firmicutes* and *Bacteroidetes* between CPHs and RPHs (Figure 5). Thus, different diets between captive and wild animals might be one of the factors influencing the composition of their gut microbes [54,55]. 

These results have also been proved in previous function analysis [44], which showed that the reintroduced Przewalski’s horses harbored bacteria with greater metabolic functional potential, which mainly participated in the functions of “energy production and conversion”, “secondary metabolites biosynthesis, transport and catabolism”, “carbohydrate transport and metabolism”, “metabolism of terpenoids and polyketides” and “drug resistance: antimicrobial”. In contrast, only genes related to the function of “cell growth and death” were significantly enriched in the captive Przewalski’s horses.

**Figure 6:**
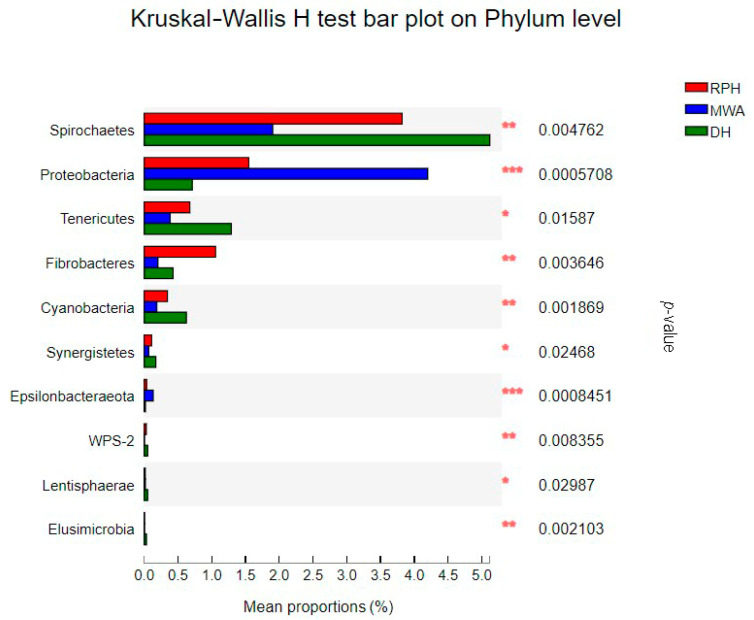
The bar plot shows the bacterial phylum with significant differences among RPH, DH and MWA. *p < 0.05, **p < 0.01 and ***p < 0.001.

In addition, the results showed that *Spirochaetes* (3.81%), *Kiritimatiellaeota* (2.98%), *Proteobacteria* (1.57%) and *Fibrobacteres* (1.04%) had relative abundance at 0 order of magnitude in RPHs (Figure 4). Among these phyla, *Proteobacteria* had significant differences among the RPHs, DHs and MWAs in the Kruskal–Wallis H test (Figure 6). A previous study has suggested that *Proteobacteria* are a signature for the dysbiosis of the microbial community and have implications for disease, and the pattern of low intestinal bacterial diversity with a dominance of *Proteobacteria* was also commonly observed in undernourished children [56]. Our study discovered that *Proteobacteria* had significant differences among sympatric RPHs, DHs and MWAs, because these three equine animals were infected by parasitic diseases [57]. A similar pattern of low intestinal bacterial diversity with a high abundance of *Proteobacteria* was also observed in an MWA population, which makes us suspect that a natural diet has a lower nutritional value than the artificial diet in PHBC. 

Other phyla with relative abundance lower than 0 order of magnitude in RPHs are *Tenericutes*, *Patescibacteria*, *Cyanobacteria*, *Verrucomicrobia*, *Actinobacteria*, *Synergistetes*, *Epsilonbacteraeota*, WPS-2, unclassified_k__norank_d__Bacteria, *Armatimonadetes*, *Lentisphaerae*, *Planctomycetes* and *Elusimicrobia Chlamydiae* and *Chloroflexi* were not found in RPHs, and the former was also not found in MWAs (Supplementary Table S2).

**Figure 7:**
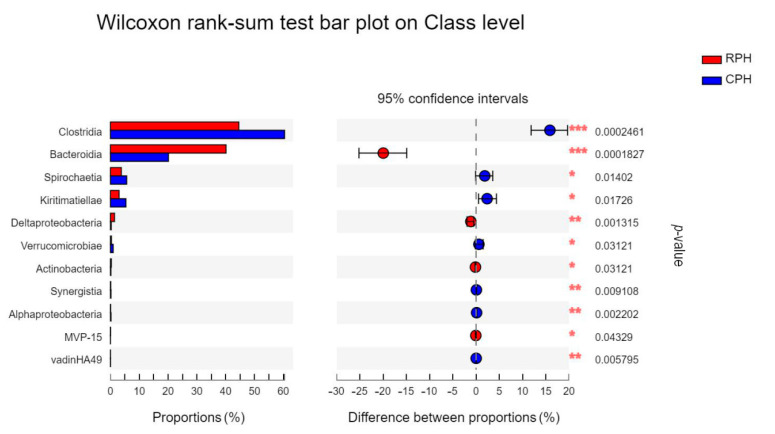
The bar plot shows the bacterial class with significant differences among the CPH and RPH. *p < 0.05, **p < 0.01 and ***p < 0.001.

**Figure 8:**
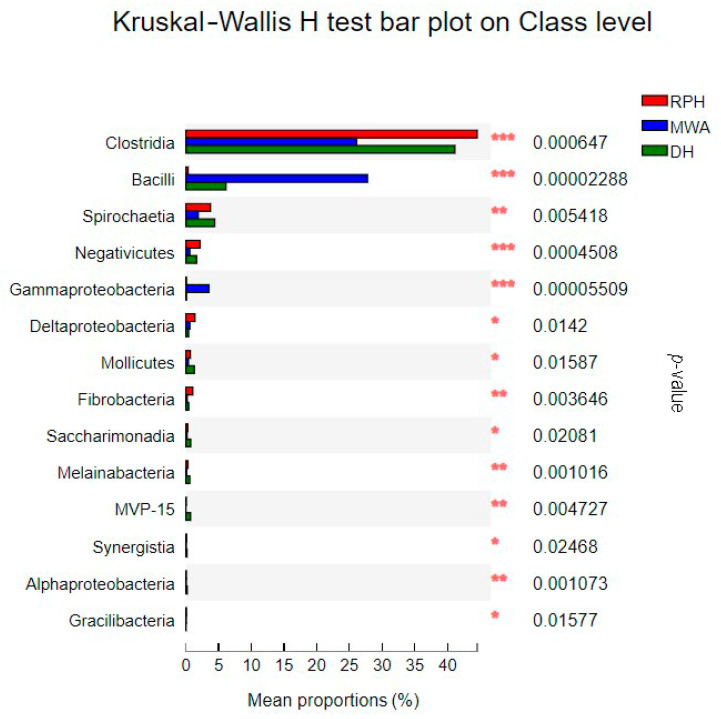
The bar plot shows the bacterial class with significant differences among RPH, DH and MWA. *p < 0.05, **p < 0.01 and ***p < 0.001.

At class level, the dominant two classes were *Clostridia* and *Bacteroidia,* which had relative abundance at 1 order of magnitude. The *Clostrida* and *Bacteroidia* had significant differences between CPHs and RPHs (Figure 7), and *Clostridia* displayed significant differences among the RPHs, DHs and MWAs (Figure 8). There are 11 classes that displayed relative abundance at 0 order of magnitude in RPHs, which were *Kiritimatiellae*, *Spirochaetia*, *Deltaproteobacteria*, *Negativicutes*, *Erysipelotrichia*, *Fibrobacteria*, *Verrucomicrobiae*, *Mollicutes*, *Bacilli*, *Saccharimonadia* and *Melainabacteria* (Supplementary Table S3). The other 19 classes had relative abundance lower than 0 order of magnitude (Supplementary Table S3). Among them, classes of *Bacilli*, *Negativicutes* and *Gammaproteobacteria* had significant differences among the RPHs, DHs and MWAs (Figure 8). 

**Figure 9:**
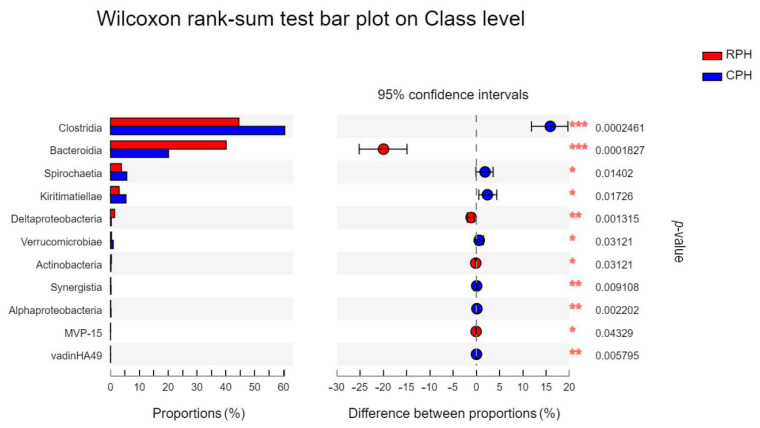
The bar plot shows the bacterial order with significant differences among the CPH and RPH. *p < 0.05, **p < 0.01 and ***p < 0.001.

**Figure 10:**
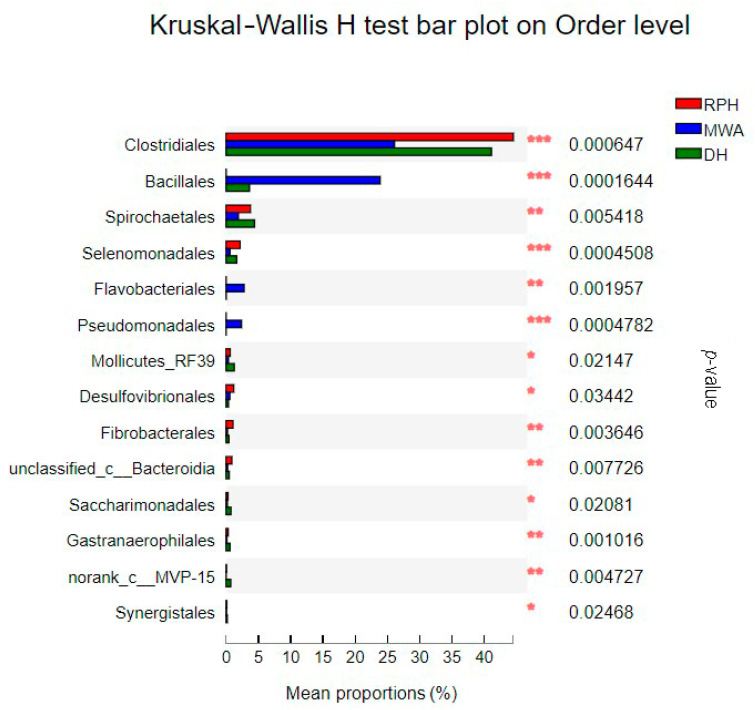
The bar plot shows the bacterial order with significant differences among RPH, DH and MWA. *p < 0.05, **p < 0.01 and ***p < 0.001.

The dominant orders in all samples were *Clostridiales* (CPHs: 60.46%; RPHs: 44.72%; DHs: 41.12%; MWAs: 25.80%) and *Bacteroidales* (CPHs: 18.47%; RPHs: 39.00%; DHs: 36.35%; MWAs: 30.19%), which belong to the classes of *Clostridia* and *Bacteroidia*, respectively. Six orders of *Spirochaetales*, WCHB1-41, *Selenomonadales*, *Desulfovibrionales*, *Erysipelotrichales* and *Fibrobacterales* had relative abundance at 0 order of magnitude in RPHs. Furthermore, the relative abundance of 60 orders was lower than 0 order of magnitude (Supplementary Table S4). Orders of *Clostridiales* and *Bacteroidales* had significant differences between CPHs and RPHs (Figure 9), and orders of *Clostridiales*, *Bacillales*, *Selenomonadales*, *Pseudomonadales* had significant differences among the RPHs, DHs and MWAs (Figure 10). 

**Figure 11:**
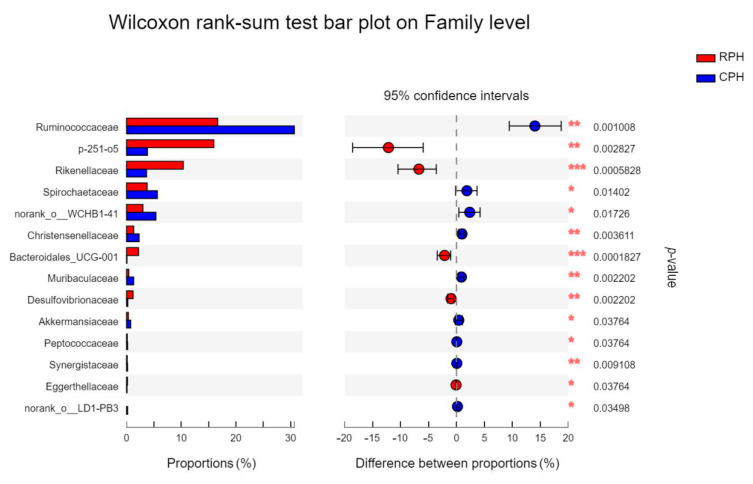
The bar plot shows the bacterial family with significant differences among the CPH and RPH. *p < 0.05, **p < 0.01 and ***p < 0.001.

**Figure 12:**
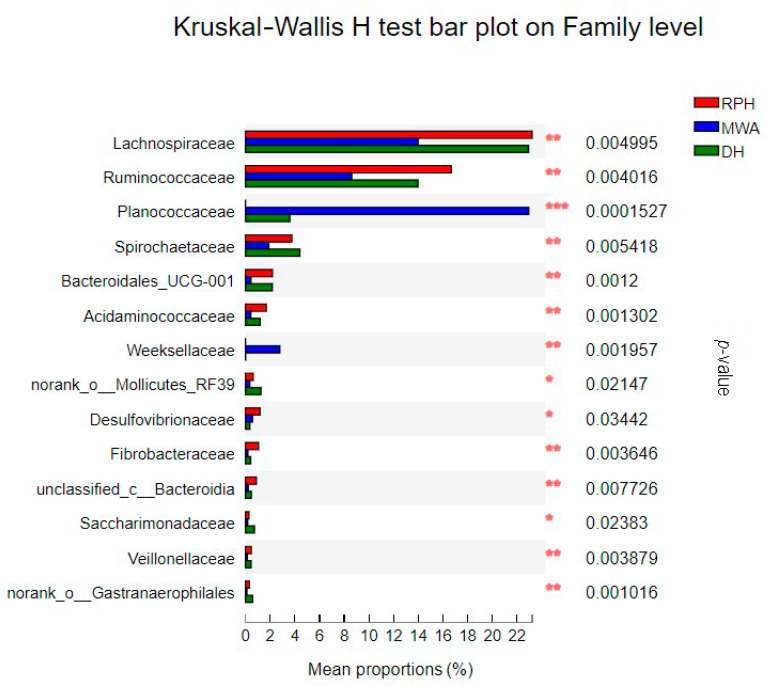
The bar plot shows the bacterial family with significant differences among RPH, DH and MWA. *p < 0.05, **p < 0.01 and ***p < 0.001.

Furthermore, a total of 116 families were found in all samples. The top four families with relative abundance at 1 order of magnitude in RPHs were *Lachnospiraceae* (23.12%), *Ruminococcaceae* (16.90%), p-251-o5 (15.52%) and *Rikenellaceae* (10.56%). For the wild animal group, DHs were lower in all families except for *Rikenellaceae*, while the others were higher in DHs and MWAs (Supplementary Table S5). The top family in MWAs was *Planococcaceae,* which showed relative abundance of 23.55%, but *Planococcaceae* had only 0.058%, 0.065% and 3.76% in CPHs, RPHs and DHs, respectively (Supplementary Table S5). The Wilcoxon rank-sum test showed that families of *Rikenellaceae* and *Bacteroidales*_UCG-001 had significant differences between CPHs and RPHs (Figure 11). The Kruskal–Wallis H test showed that the family of *Planococcaceae* had significant differences among the RPHs, DHs and MWAs (Figure 12). 

The dominant classes and orders were consistent in all samples, but the dominant bacteria differ at family levels. It can be inferred that captive animals and wild animals have different bacterial community patterns under the level of family. For the significantly changed bacterial taxa, the *Clostridia*, *Bacilli*, *Negativicutes*, *Clostridiales*, *Bacillales*, *Selenomonadales*, *Planococcaceae*, *Solibacillus* and *Lachnospiraceae*_AC2044_group were classified into *Firmicutes*, while *Gammaproteobacteria* and *Pseudomonadales* were classified into *Proteobacteria*. Although RPHs, DHs and MWAs lived in the same region with the same climate conditions, their health status and range of activities were different, which will lead to different feeding habits. According to previous studies, donkeys have a wider range of food than horses in the wild [31,34,58]. Thus, the differences in intestinal bacterial composition among sympatric wild animals might be related to the different feeding habits. Furthermore, we speculate that equine animals in the KNR are facing huge potential disease risks and environmental pressures. Currently, MWAs are already showing signs of undernourishment, so it is necessary to apply dietary interventions for RPHs in case a similar bacterial community pattern appears over time in the wild. In addition, the Wilcoxon rank-sum test showed that *Armatimonadetes* together with *Firmicutes* and *Bacteroidetes* were associated with the transition from captive to wild (Figure 5). As mentioned before, the functions of *Firmicutes* and *Bacteroidetes* are related to food digestion [52], so we speculate that *Armatimonadetes* may have the same function as *Firmicutes* and *Bacteroidetes*, which has not been reported before. Moreover, *Clostrida*, *Clostridiales* and [*Eubacterium*]_*coprostanoligenes* are classified into *Firmicutes*, while *Bacteroidia*, *Bacteroidales*, *Rikenellaceae* and *Bacteroidales*_UCG-001 are classified into *Bacteroidetes*. Thus, the differences in intestinal microbiota between captive and reintroduced Przewalski’s horses may be related to the change in environmental conditions, which is mainly dietary. 

## 4. Conclusions

Reintroduced animals released into new habitats have to face multiple pressures from the natural environment. In this study, the intestinal bacterial communities of captive, reintroduced and wild equine animals were documented, which has provided important information for understanding and managing the reintroduced animals. From the perspective of intestinal bacteria, released Przewalski’s horses have not fully adapted to the wild environment, even though their behavior has become similar to that of wild animals. Management and human interventions are necessary in the early stages after reintroduced animals are released into the wild. The adaptation and development of reintroduced animals to a new environment with the help of intestinal bacteria is also a long-term process. However, a limitation of this study is that the link between our observations and conservation outcomes might be indirect. Thus, an experiment on population change (decline or increase) should be conducted in the future. 

## Figures and Tables

**Table 1:** The sex information of sampled horses.

Sex	CPH	RPH	MWA	DH
Female	CPH1, CPH4, CPH5, CPH8, CPH10	RPH1, RPH2, RPH5, RPH7, RPH10	MWA1, MWA3, MWA4, MWA7, MWA10	DH2, DH4, DH5, DH6, DH9
Male	CPH2, CPH3, CPH6, CPH7, CPH9	RPH3, RPH4, RPH6, RPH8, RPH9	MWA2, MWA5, MWA6, MWA8, MWA9	DH1, DH3, DH7, DH8, DH10

## Data Availability

The sequencing data generated in our study have been deposited in the SRA database and the BioProject ID is PRJNA624720.

## Supplementary Materials

The following supporting information can be downloaded at: https://www.mdpi.com/article/10.3390/ani12202874/s1, Table S1: Relative abundance (%) of *Firmicutes* and *Bacteroidetes* was identified in 40 fecal samples of 4 equine animals; Table S2: Relative abundance (%) of phylum was identified in 40 fecal samples of 4 equine animals; Table S3: Relative abundance (%) of class was identified in 40 fecal samples of 4 equine animals; Table S4: Relative abundance (%) of order was identified in 40 fecal samples of 4 equine animals; Table S5: Relative abundance (%) of family was identified in 40 fecal samples of 4 equine animals.

## References

[1] King S.R.B., Boyd L., Zimmermann W., Kendall B. (2015). *Equus ferus* ssp. *Przewalskii*. IUCN 2016.

[2] Mohr E. (1972). The Asiatic Wild Horse.

[3] Zhang H., Sun L., Cao J. (2002). The Breeding Status of Captive *Przewalskii horse* in Xinjiang. Chin. J. Zool..

[4] Chu H., Jiang Z.G., Lan W., Wang C., Tao Y.S., Jiang F. (2008). Dietary overlap among *Kulan Equus hemionus*, Goitered gazelle *Gazella subgutturosa* and livestock. Acta. Zool. Sin..

[5] Cao J., Hu D., Chen J., Zhang H. (2007). A preliminary observation on the behavioral changes of released *Prezwalski’s horses* in rewilding process. J. Xinjiang Norm. Univ. Nat. Sci. Ed..

[6] Chen J. (2008). Utilization of Food, Water and Space by Released *Przewalski horse* (*Equus przewalski*) with Reference to Survival Strategies Analysis. J. Beijing For. Univ..

[7] Wang J., Hu D., Li K., Chen J. (2009). Study on the behavior and activity budgets of reintroduced female *Przewalski horse* in lactation period. J. Xinjiang Norm. Univ. Nat. Sci. Ed..

[8] Zhang F., Hu D., Li K., Cao J., Chen J., Waltrau Z. (2009). The agonistic behavior and hierarchical formation of the *Equus przewalskii* herd in the individual coalition and initial releasing period. Chin. J. Zool..

[9] Yu X., Hu D., Tang Y., Ji S., Zhang Y., Liu G. (2011). Physiological response of behavior and stress in breeding group of *Przewalskii horse* during releasing process. China Anim. Husb. Vet. Med..

[10] Huang X., Ji S., Zhang Y., Cao J., Chu H., Li K., Hu D. (2012). Change of released *Equus przewalskii* family and the implication to their management in initial period. Sichuan J. Zool..

[11] Meng Y., Hu D., He D., Chen J., Zhang F. (2009). The breeding status of the released *Przewalski horses* in Xinjiang, China. Bull. Biol..

[12] Huang H., Chu H., Cao J., Bu L., Hu D., Zhang D., Li K. (2017). Distribution of *Gasterophilus* (*Diptera*, *Gasterophilidae*) myiasis foci in arid desert steppe: A case study of Kalamaili Mountain Ungulate Nature Reserve. Scien. Silva. Sin..

[13] Liu G., Shafer A.B., Zimmermann W., Hu D., Wang W.T., Chu H.J., Cao J., Zhao C.X. (2014). Evaluating the reintroduction project of *Przewalski*’s *horse* in China using genetic and pedigree data. Biol. Conserv..

[14] Hu D., Chao Y., Zhang B., Wang C., Qi Y., Ente M., Zhang D., Li K., Mok K.M. (2021). Effects of *Gasterophilus pecorum* infestation on the intestinal microbiota of the rewilded *Przewalski*’s *horses* in China. PLoS ONE.

[15] Berry D., Kuzyk O., Rauch I., Heider S., Schwab C., Hainzl E., Decker T., Müller M., Strobl B., Schleper C. (2015). Intestinal microbiota signatures associated with inflammation history in mice experiencing recurring colitis. Front. Microbiol..

[16] Xiong J., Wang K., Wu J., Qiuqian L., Yang K., Qian Y., Zhang D. (2015). Changes in intestinal bacterial communities are closely associated with shrimp disease severity. Appl. Microbiol. Biot..

[17] Sadet-Bourgeteau S., Julliand V. (2012). The diversity of the microbial ecosystem in the equine digestive tract. INRA. Prod. Anim..

[18] Sadet-Bourgeteau S., Julliand V. (2010). Equine microbial gastro-intestinal health. EAAP Sci. Ser..

[19] van Leeuwen P., Mykytczuk N., Mastromonaco G.F., Schulte-Hostedde A. (2020). Effects of captivity, diet, and relocation on the gut bacterial communities of white-footed mice. Ecol. Evol..

[20] Sjögren Y.M., Tomicic S., Lundberg A., Böttcher M.F., Björkstén B., Sverremark-Ekström E., Jenmalm M.C. (2009). Influence of early gut microbiota on the maturation of childhood mucosal and systemic immune responses: Gut microbiota and immune responses. Clini. Exp. Allergy..

[21] El Aidy S., Van Den Bogert B., Kleerebezem M. (2015). The small intestine microbiota, nutritional modulation and relevance for health. Curr. Opin. Biotech..

[22] Michail S., Lin M., Frey M.R., Fanter R., Wai D., Cleary J.G., Hilbush B. (2015). Altered gut microbial energy and metabolism in children with non-alcoholic fatty liver disease. Fems. Microbiol. Ecol..

[23] Murphy E.A., Velazquez K.T., Herbert K.M. (2015). Influence of high-fat-diet on gut microbiota: A driving force for chronic disease risk. Curr. Opin. Clin. Nutr. Metab. Care..

[24] Boulangé C.L., Neves A.L., Chilloux J., Nicholson J.K., Dumass M.E. (2016). Impact of the gut microbiota on inflammation, obesity, and metabolic disease. Genome. Med..

[25] Li X., Watanabe K., Kimura I. (2017). Gut microbiota dysbiosis drives and implies novel therapeutic strategies for diabetes mellitus and related metabolic diseases. Front. Immunol..

[26] Sun M.F., Shen Y.Q. (2018). Dysbiosis of gut microbiota and microbial metabolites in Parkinson’s Disease. Ageing. Res. Rev..

[27] Quiroga-González C., Cardenas L.A.C., Ramírez M., Reyes A., González C., Stevenson P.R. (2021). Monitoring the variation in the gut microbiota of captive woolly monkeys related to changes in diet during a reintroduction process. Sci. Rep..

[28] Russell W.M.S., Burch R. (1959). The Principles of Humane Experimental Technique.

[29] Wu H., Chu H., Wang Y., Ma J., Ge Y. (2014). Monitoring activity rhythms of Equus hemionus at watering holes by camera traps in Mount Kalamaili Ungulate Nature Reserve, Xinjiang. Biodivers. Sci..

[30] Sen Z., Qing C., Keremu A., Liu S., Zhang Y., Hu D. (2017). Food patch particularity and forging strategy of reintroduced Przewalski’s horse in North Xinjiang, China. Turk. J. Zool..

[31] Meng Y., Hu D., Chen J. Study on feeding source plants and strategy of wild *Przewalskii horse*. Proceedings of the Fourth National Symposium on Wildlife Ecology and Resource Conservation 2007.

[32] Ge Y., Liu C., Chu H., Tao Y. (2003). Present situation of the *Equus hemionus* resources in the Karamori Mountain Nature Reserve, Xinjiang. Arid Zone Res..

[33] Ji S. (2013). Non-Invasive Study of the Behavioral and Physiological Ecology Adaptation in Captive *Przewalski’s horse* (*Equus ferus przewalskii*). Ph.D. Thesis.

[34] Chen J., Hu D., Li K., Cao J., Meng Y., Cui Y.Y. (2008). The diurnal feeding behavior comparison between the realeased and captive adult female *Przewalski’s horse* (*Equus przewalskii*) in summer. Acta. Ecol. Sin..

[35] Riquelme J., Cazanga V., Jeldres J., Pérez R. (2018). Pharmacokinetics of ivermectin in sheep following pretreatment with Escherichia coli endotoxin. J. Vet. Pharmacol. Ther..

[36] Yu M., Jia H., Zhou C., Yang Y., Zhao Y., Yang M., Zou Z. (2017). Variations in gut microbiota and fecal metabolic phenotype associated with depression by 16S rRNA gene sequencing and LC/MS-based metabolomics. J. Pharmaceut. Biomed..

[37] Quast C., Pruesse E., Yilmaz P., Gerken J., Schweer T., Yarza P., Peplies J., Glockner F.O. (2012). The SILVA ribosomal RNA gene database project: Improved data processing and web-based tools. Nucleic. Acids. Res..

[38] Salem S.E., Maddox T.W., Berg A., Antczak P., Ketley J.M., Williams N.J., Archer D.C. (2018). Variation in faecal microbiota in a group of horses managed at pasture over a 12-month period. Sci. Rep..

[39] Lin H., Peddada S.D. (2020). Analysis of microbial compositions: A review of normalization and differential abundance analysis. NPJ. Biofilms. Microbi..

[40] Caporaso J.G., Kuczynski J., Stombaugh J., Bittinger K., Bushman F.D., Costello E.K., Fierer N., Peña A.G., Goodrich J.K., Gordon J.I. (2010). QIIME allows analysis of high-throughput community sequencing data. Nat. Methods..

[41] Ihaka R., Gentleman R. (1996). R: A language for data analysis and graphics. J. Comput. Graph. Stat..

[42] Deng G., Zha Y., Zhang G., Wang Y., Li Y., Peng X., Zhou H., Liu Y. (2014). Comparison of human and animal fecal microbiota with Illumina sequencing of 16S rRNA tags. Ecologic. Sci..

[43] Li Y., Zhang K., Liu Y., Li K., Hu D., Wronski T. (2019). Community composition and diversity of intestinal microbiota in captive and reintroduced *Przewalski’s horse* (*Equus ferus przewalskii*). Front. Microbiol..

[44] Tang L., Li Y., Srivathsan A., Guo Y., Li K., Hu D., Zhang D. (2020). Gut microbiomes of endangered *Przewalski’s horse* populations in short-and long-term captivity: Implication for species reintroduction based on the soft-release strategy. Front. Microbiol..

[45] Chen J., Hu D., Cao J., Lv Q., Men Y. (2008). A preliminary report on the summer water resources used by *Equus przewalskii*. J. Xinjiang Norm. Univ. Nat. Sci. Ed..

[46] Liu R., Shi J., Shultz S., Guo D., Liu D. (2021). Fecal bacterial community of allopatric *Przewalski’s gazelles* and their sympatric relatives. Front. Microbiol..

[47] Zhao Y., Li B., Bai D., Huang J., Shiraigo W., Yang L., Zhao Q., Ren X., Wu J., Bao W. (2016). Comparison of fecal microbiota of Mongolian and thoroughbred horses by high-throughput sequencing of the V4 Region of the 16S rRNA gene. Asian–Austral. J. Anim. Sci..

[48] Guan Y., Yang H., Han S., Feng L., Wang T., Ge J. (2017). Comparison of the gut microbiota composition between wild and captive sika deer (*Cervus nippon* hortulorum) from feces by high-throughput sequencing. AMB Express.

[49] Zhang M., Shi M., Fan M., Xu S., Li Y., Zhang T., Cha M., Liu Y., Guo X., Chen Q. (2018). Comparative analysis of gut microbiota changes in Père David’s deer populations in Beijing Milu Park and Shishou, Hubei province in China. Front. Microbiol..

[50] Xu J., Bjursell M.K., Himrod J., Deng S., Carmichael L.K., Chiang H.C., Hooper L.V., Gordon J.I. (2003). A genomic view of the human-Bacteroides thetaiotaomicron symbiosis. Science.

[51] Bäckhed F., Ley R.E., Sonnenburg J.L., Peterson D.A., Gordon J.I. (2005). Host-bacterial mutualism in the human intestine. Science.

[52] Chen S., Cheng H., Wyckoff K.N., He Q. (2016). Linkages of Firmicutes and Bacteroidetes populations to methanogenic process performance. J Ind. Microbiol. Biot..

[53] Biddle A., Tomb J.F., Fan Z. (2018). Microbiome and blood analyte differences point to community and metabolic signatures in lean and obese horses. Front. Vet. Sci..

[54] Schwab C., Cristescu B., Northrup J.M., Stenhouse G.B., Ganzle M. (2011). Diet and environment shape fecal bacterial microbiota composition and enteric pathogen load of grizzly bears. PLoS ONE.

[55] Navarrete P., Magne F., Araneda C., Fuentes P., Barros L., Opazo R., Espejo R., Romero J. (2012). PCR-TTGE analysis of 16S rRNA from rainbow trout (Oncorhynchus mykiss) gut microbiota reveals host-specific communities of active bacteria. PLoS ONE.

[56] Shin N.R., Whon T.W., Bae J.W. (2015). Proteobacteria: Microbial signature of dysbiosis in gut microbiota. Trends. Biotechnol..

[57] Huang H., Zhang B., Chu H., Zhang D., Li K. (2016). *Gasterophilus* (*Diptera*, *Gasterophilidae*) infestation of equids in the Kalamaili nature Reserve, China. Parasite.

[58] Xu W., Yang W., Qiao J. (2009). Food habits of Kulan (*Equus hemionus*) in Kalamaili Mountain Nature Reserve, Xinjiang, China. Acta Theriologica Sin..

